# The C_2_H_2_-Type Transcription Factor ZfpA, Coordinately with CrzA, Affects Azole Susceptibility by Regulating the Multidrug Transporter Gene *atrF* in Aspergillus fumigatus

**DOI:** 10.1128/spectrum.00325-23

**Published:** 2023-06-15

**Authors:** Yeqi Li, Mengyao Dai, Ling Lu, Yuanwei Zhang

**Affiliations:** a Jiangsu Key Laboratory for Microbes and Functional Genomics, Jiangsu Engineering and Technology Research Centre for Microbiology, College of Life Sciences, Nanjing Normal University, Nanjing, China; Universidade de Sao Paulo

**Keywords:** drug susceptibility, negative regulator, ABC transporter, fungal pathogen, *Aspergillus fumigatus*

## Abstract

The incidence of invasive aspergillosis caused by Aspergillus fumigatus has risen steadily over the past few decades due to the limited effective treatment options and the emergence of antifungal-resistant isolates. In clinic-isolated A. fumigatus, the azole resistance mechanism is primarily caused by mutations of the drug target and/or overexpression of drug efflux pumps. However, knowledge about how drug efflux pumps are transcriptionally regulated is limited. In this study, we found that loss of a C_2_H_2_ transcription factor ZfpA (zinc finger protein) results in the marked upregulation of a series of drug efflux pump-encoding genes, especially *atrF*, which contributes to azole drug resistance in A. fumigatus. CrzA is a previously identified positive transcription factor for genes of drug efflux pumps, and ZfpA transcriptionally inhibits expressions of drug efflux pumps in a CrzA-dependent way. Under the treatment of azoles, both ZfpA and CrzA transfer to nuclei and coregulate the expression of multidrug transporters and then keep normal drug susceptibility in fungal cells. Findings in this study demonstrated that ZfpA is not only involved in fungal growth and virulence potential but also negatively regulates antifungal drug susceptibility.

**IMPORTANCE** Conserved across all kingdoms of life, ABC transporters comprise one of the largest protein families. They are associated with multidrug resistance, affecting aspects such as resistance to antimicrobials or anticancer drugs. Despite the importance of ABC transporters in multidrug resistance, the understanding of their regulatory network is still limited in A. fumigatus. Here, we found that the loss of the transcription factor ZfpA induces the expression of the ABC transporter gene *atrF*, altering azole susceptibility in A. fumigatus. ZfpA, coordinately with CrzA, affects the azole susceptibility by regulating the expression of the ABC transporter gene *atrF*. These findings reveal the regulatory mechanism of the ABC transporter gene *atrF* in A. fumigatus.

## INTRODUCTION

Aspergillus fumigatus, one of the most important airborne mold pathogens and allergens, can cause aspergillosis (1–3). According to the statistics by the Global Action Fund for Fungal Infections (GAFFI), more than 3 million people have invasive or chronic infections, leading to over 600,000 deaths every year (2). Despite the population of fungal infections continuously increasing, the availability of antifungal drugs is still limited (4, 5). Only three classes of drugs are currently recommended to treat aspergillosis, including azoles for the primary therapeutic purpose of fungal infectious diseases, amphotericin B and echinocandins for salvage therapy (5, 6). Further worsening the outlook for antifungal therapy, a steady increase in azole resistance in A. fumigatus has been a major clinical complication in the treatment of fungal disease, and the mortality rate of individuals infected with a resistant isolate exceeds 80% (6, 7). Thus, unraveling the molecular mechanisms of drug resistance and developing new antifungal drugs to combat invasive aspergillosis are urgently needed.

Mutations in the *cyp51A* gene locus, which encodes the target enzyme lanosterol-α-14-demethylase of azole drugs, have been frequently reported in azole-resistant strains of A. fumigatus (8). Apart from alterations in the drug target Cyp51, activation of drug efflux pumps and induction of cellular stress responses also cause azole resistance (7, 9, 10). Activation of drug efflux pumps in resistant strains usually is mediated by mutations, which cause high expression levels of these pumps (11, 12). There are two main families of drug efflux pumps, the ATP binding cassette (ABC) and major facilitator superfamily (MFS) pumps, of which the genes are highly redundant in A. fumigatus (49 ABC and nearly 278 MFS classes) (13–15). These membrane proteins are important for the protection of fungi against antifungals by extruding cytotoxic drugs from cells, thus leading to reduced drug accumulation (12). According to phylogenetic analysis, A. fumigatus contains an ABC-G subfamily that harbors 12 ABC transporters, including the previously characterized Cdr1B, AbcA, AtrF, and AtrI (9, 10, 16, 17). Previous work found that the overexpression of *atrF* is critical for azole resistance in clinical A. fumigatus isolates (10). Therefore, a better understanding of the regulatory mechanism of the ABC transporter gene *atrF* will illuminate new approaches to control azole resistance. In the pathogenic yeast Candida glabrata, ABC transporter Snq2 (an ortholog of A. fumigatus AtrF) is regulated by the zinc cluster transcriptional regulator Pdr1 (18). In the filamentous fungus Aspergillus flavus, the gain of function of the bZIP transcription factor Yap1 causes the upregulation of *atrF* (19). However, the regulatory mechanism of AtrF is still unclear in A. fumigatus.

The conserved calcium signaling pathway plays a crucial role in regulating multiple cell functions ranging from growth, development, fertility, stress response, and virulence in fungi (20, 21). Notably, it has been reported that azole resistance in fungal pathogens is tightly related to the regulation of the calcium signaling pathway (20, 21). In our previous work, we found that the abnormal activation of transcription factor CrzA in azole-resistant strains induces high expressions of a series of drug efflux-encoding genes, including *atrF*, but electrophoretic mobility shift assays (EMSAs) do not detect the binding of CrzA in the predicted motif of *atrF* promoter (22). Thus, we speculate that CrzA may indirectly regulate the expression of *atrF* by affecting some unknown targets. Based on the publicly available chromatin immunoprecipitation sequencing (ChIP-seq) data of CrzA in A. fumigatus (23), we found eight putative CrzA binding transcription factors, including the C_2_H_2_ transcription factor zinc finger protein A (ZfpA). ZfpA was first found in the transcriptome of A. fumigatus exposed to voriconazole (17). Malavazi and colleagues found that the deletion of *zfpA* causes the increased MIC of itraconazole and decreased MIC of caspofungin in A. fumigatus (24). Moreover, Niu and her colleagues found that ZfpA overexpression induces hyperbranched hyphae and cell wall chitin. Conversely, the hyphae of *zfpA* deletion show reduced branching, septation, and cell wall chitin (25). In addition, ZfpA is critical for A. fumigatus to respond to innate immune cells during the infection and also remodels the construction of cell wall to decrease the efficacy of antifungal drugs (26). Despite these valuable observations of ZfpA, the understanding of the mechanism by which ZfpA influences azole susceptibility remains incomplete.

In this study, we found that the loss of a C_2_H_2_ transcription factor ZfpA causes the upregulation of the drug efflux pump-encoding gene *atrF*, which contributes to its decreased azole susceptibility in A. fumigatus. Furthermore, we found that the upregulation of drug efflux pump genes in the *zfpA* deletion strain is dependent on the expression of transcription factor CrzA. The results of our study suggest that the transcription factor ZfpA, coordinately with CrzA, affects azole susceptibility by regulating the expression of the multidrug transporter gene.

## RESULTS

### Identification of putative CrzA downstream transcription factors involved in azole susceptibility.

**FIG 1:**
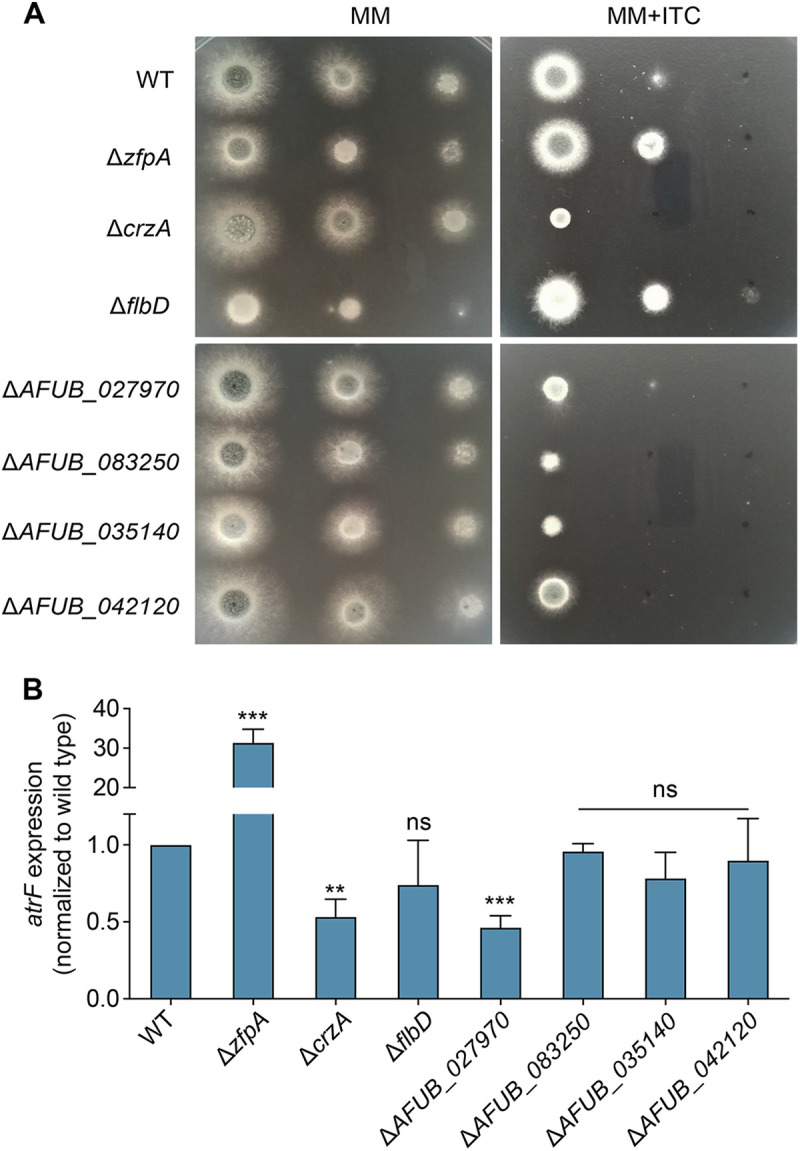
Azole susceptibility and mRNA expression of *atrF* in putative CrzA-targeted transcription factor deletion mutants. (A) The indicated strains were spotted onto the solid medium at 37°C for 2 days (without drug) or 3 days (with drug; ITC, 0.1 μg/mL). (B) Expression changes of mRNA identified in the indicated strains by qRT-PCR for the drug efflux pump-encoding gene *atrF*. Statistical significance was determined using one-way analysis of variance (ANOVA) for multiple comparisons. ns, not significant; **, *P < *0.01; ***, *P < *0.001.

Our previous study demonstrated that CrzA is a positive transcription factor indirectly required for the expression of the drug efflux pump-encoding gene *atrF*, which contributes to azole resistance. To further explore whether there are transcription factors targeted by CrzA that may affect the expression of drug efflux pump *atrF*, ChIP-seq data for CrzA-targeted proteins were analyzed (23). Eight genes had the possible DNA binding activity, including *crzA* itself, as shown in Table 1. To verify whether these transcription factors are truly associated with azole susceptibility, we generated deletion mutants of these genes and detected phenotypes of these null mutants under the itraconazole-treated cultural conditions. As shown in Fig. 1A, Δ*crzA*, Δ*AFUB_027970*, Δ*AFUB_042120*, Δ*AFUB_35140*, and Δ*AFUB_083250* showed increased susceptibility to itraconazole (ITC) compared to the parental wild-type strain. In contrast, Δ*zfpA* and Δ*flbD* mutants exhibited significantly reduced susceptibility to azole.

**TABLE 1:** The putative transcription factors regulated by CrzA from ChIP-seq data

Gene	Annotation
AFUB_007280 (*crzA*)	C_2_H_2_-type zinc finger transcription factor involved in calcium ion homeostasis and regulation of conidial germination, hyphal growth, and virulence
AFUB_082490 (*zfpA*)	Putative C_2_H_2_ zinc finger transcription factor, transcript induced by voriconazole and calcium
AFUB_025730 (*ndtA*)	Has domain(s) with predicted DNA binding activity, DNA-dependent meiosis-specific transcription factor; activates middle sporulation genes; competes with Sum1p for binding to promoters containing middle sporulation elements
AFUB_003630 (*flbD*)	Myb family transcription factor has role in positive regulation of conidium formation and localizes to nucleus
AFUB_027970	HLH transcription factor, activator that forms a heterodimer with Ino2; likely regulates genes involved in phosphatidylcholine and phosphatidylinositol biosynthesis, fatty acid beta-oxidation, and peroxisome biogenesis
AFUB_035140	Has domain(s) with predicted zinc ion binding activity and intracellular localization
AFUB_042120	C_2_H_2_ finger domain protein, putative
AFUB_083250	C_2_H_2_ finger domain protein, putative

To further test whether the aforementioned genes could affect the *atrF* transcription, the mRNA level of *atrF* in these null mutants had been quantified as shown in Fig. 1B. The expression of *atrF* in Δ*flbD*, Δ*AFUB_042120*, Δ*AFUB_35140*, and Δ*AFUB_083250* mutants was similar to that in the wild-type strain. However, the expression of *atrF* in *crzA* and Δ*AFUB_027970* mutants was significantly decreased compared to that in the parental wild-type strain, suggesting both of them were required for the normal expression of *atrF*. Interestingly, deletion of *zfpA* exhibited increased expression of *atrF*, suggesting that ZfpA may affect the expression of *atrF* oppositely with the transcription factor CrzA, which may result in contrasting azole susceptibilities between the *zfpA*- and *crzA*-null mutants. We therefore further characterized the potential mechanism involved in azole susceptibility by ZfpA.

### Loss of ZfpA leads to decreased hyphal growth, azole susceptibility, and attenuated animal virulence.

**FIG 2:**
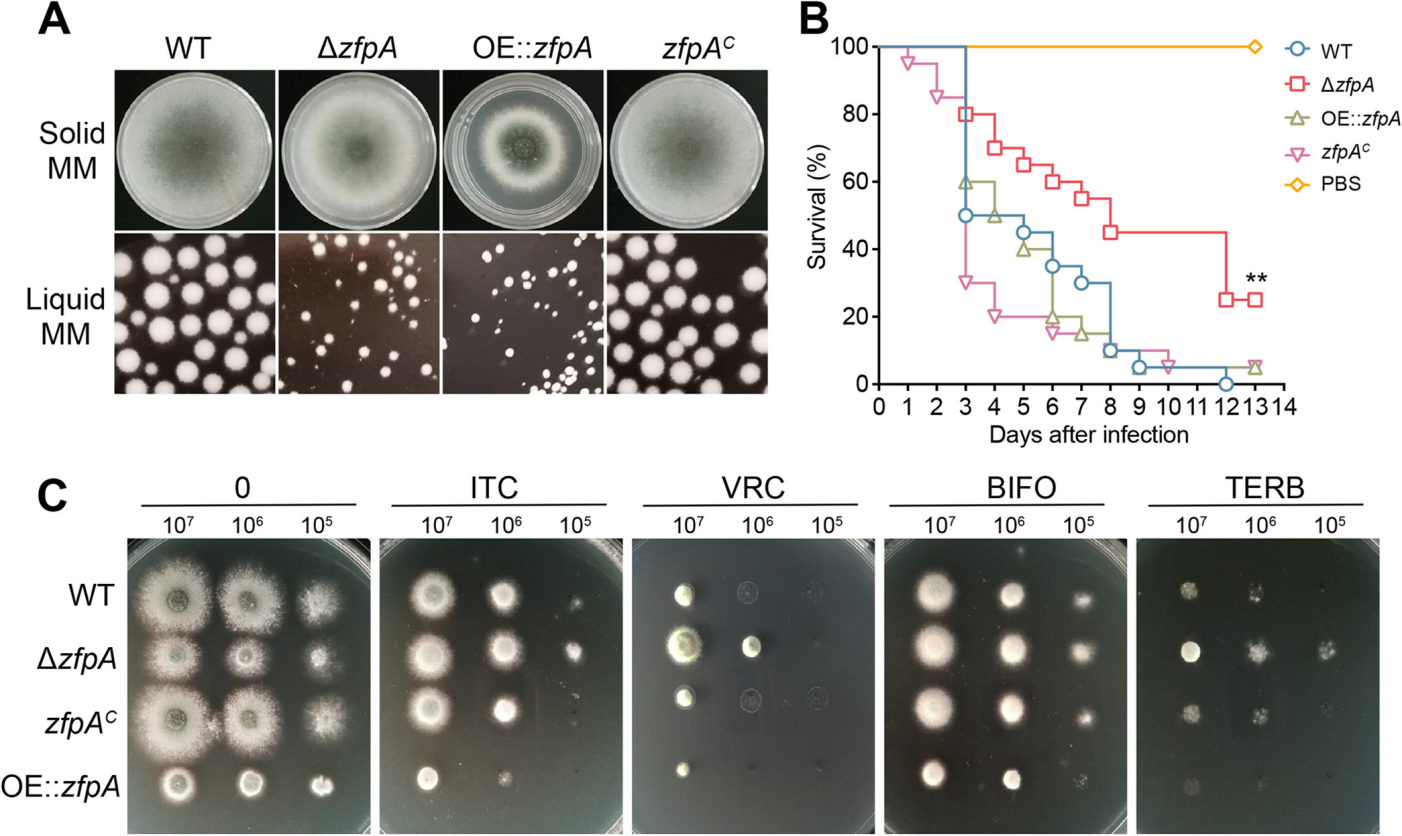
Phenotypic characterization of *zfpA* mutants. (A) Growth phenotypes of the indicated strains in the solid and liquid medium at 37°C for 2 days. (B) Survival curves of G. mellonella larvae infected with the indicated strains. PBS-injected larvae served as controls. (C) The indicated strains were inoculated onto solid medium at 37°C for 2 days (without drugs) or 3 days (with drugs). The drug concentrations were as follows: 0.1 μg/mL itraconazole (ITC), 0.1 μg/mL voriconazole (VRC), 0.5 μg/mL bifonazole (BIFO), and 0.5 μg/mL terbinafine (TERB).

According to phylogenetic analyses and homology searches using the A. fumigatus ZfpA sequences, we found that proteins homologous to ZfpA were only present in a limited number of species in Leotiomycetes (see Fig. S1 in the supplemental material). To gain insight into the function of ZfpA in A. fumigatus, we also generated a *zfpA* gene-overexpressing strain by replacing the *zfpA* native promoter with the strong constitutive Aspergillus nidulans *gpdA* promoter (Fig. S2A and B). Compared to the parental wild-type strain, the *zfpA* deletion or overexpression strain displayed dramatically reduced growth and conidiation (Fig. 2A and Fig. S2C and D). Moreover, the biomass of the *zfpA* deletion or overexpressed strain reached only 10% of that of the parental wild-type strain, suggesting that the lacking or overexpressing of ZfpA was harmful to A. fumigatus growth (Fig. S2E and Fig. S2F). The growth defect of the deletion strain was cured by reintegration of the *zfpA* gene, underlining the accuracy of the genetic manipulation (Fig. 2A). These data suggest that ZfpA is involved in vegetative growth and conidiation in A. fumigatus. To further test the functions of ZfpA *in vivo*, we analyzed the virulence potential in the wax moth Galleria mellonella. In the insect model, the *zfpA* deletion strain showed a higher survival rate of G. mellonella larvae than the parental wild-type strain (Fig. 2B), suggesting that the loss of ZfpA causes reduced virulence in A. fumigatus. However, the *zfpA* overexpression strain showed a growth defect *in vitro* but exhibited a similar virulence potential to that of the parental wild-type strain. To further examine the drug susceptibility of ZfpA, we tested the phenotypes of the *zfpA* mutants under various drug stresses and assessed the susceptibility by comparing the reduced colony diameter between minimal medium and supplemented with drugs. Consistently with previous data (24, 26), both the *zfpA* deletion and overexpression strain exhibited reduced susceptibility to azole drugs compared to the wild-type strain (Fig. 2C and Fig. S2G). These data suggest that ZfpA in A. fumigatus plays important roles in response to azole susceptibility.

### Azole antifungals, but not the other tested reagents, induce ZfpA nuclear localization.

**FIG 3:**
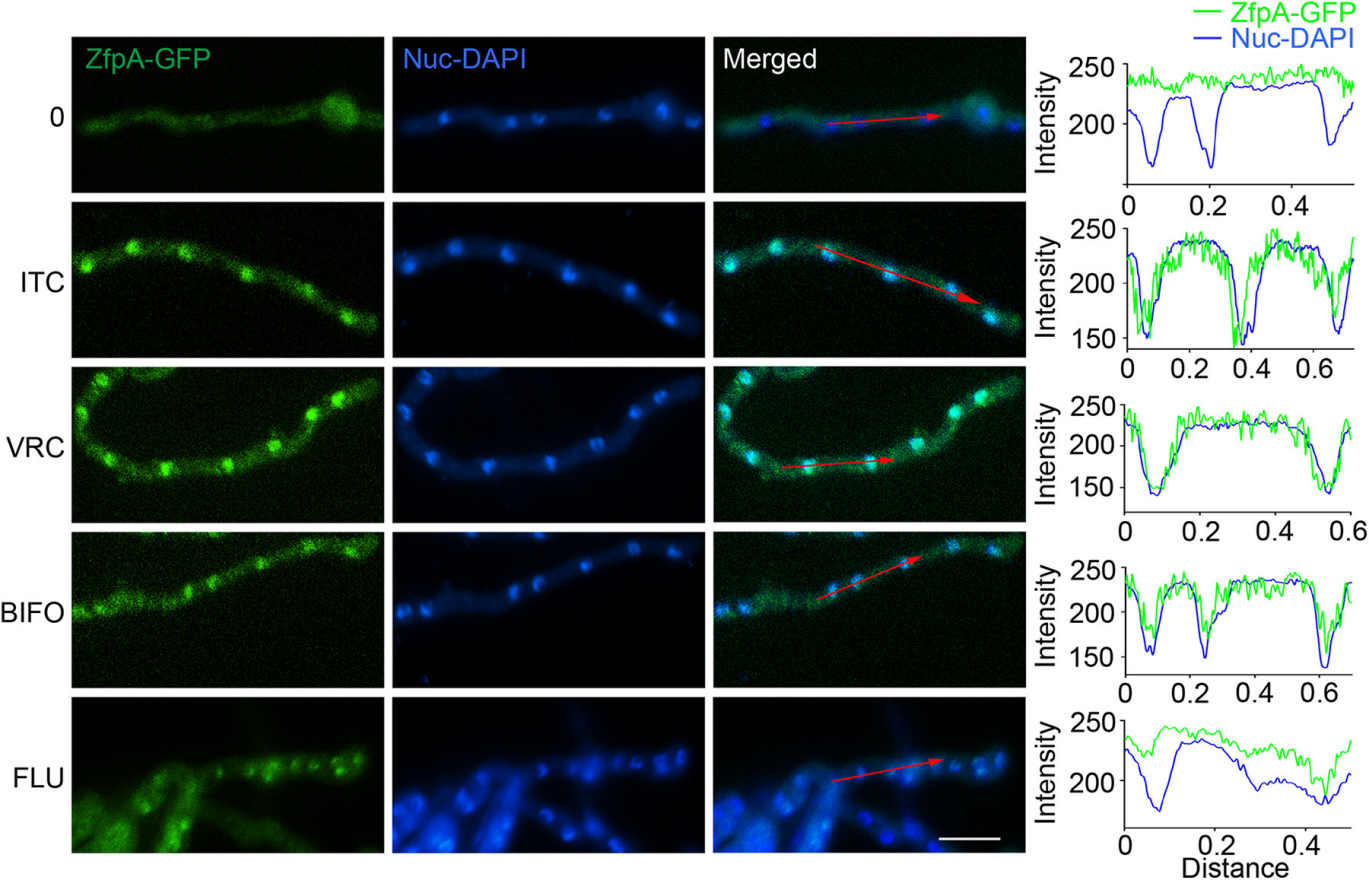
Localization of ZfpA in A. fumigatus under azole treatment. Epifluorescence microscopic images demonstrating the ZfpA-GFP distribution under untreated or treated conditions with itraconazole (ITC, 0.1 μg/mL), voriconazole (VRC, 0.1 μg/mL), bifonazole (BIFO, 0.5 μg/mL), and fluconazole (FLU, 0.5 μg/mL) for 30 min. DAPI was used as a nuclear localization signal dye to visualize the nuclei. The merged images of GFP and DAPI staining showed nuclear localization of ZfpA-GFP. The green line represents ZfpA-GFP, and the blue line represents DAPI. Distribution patterns of ZfpA-GFP and DAPI were analyzed along the red arrow lines with respect to the distance in the merged images using ImageJ software. Bars, 10 μm.

Because the transcript level of *zfpA* was changed in response to calcium or azole stimulation in a previous study (27), we reasoned that ZfpA might be a critical regulator under azole or calcium stresses. Therefore, we analyzed the cellular localization of ZfpA in response to different stimuli, including azole, calcium, and cell wall stresses. Epifluorescence microscopy studies revealed the predominant cytoplasmic localization of ZfpA-green fluorescent protein (GFP) under normal culture conditions (Fig. 3). Strikingly, once stimulated by the addition of itraconazole, a dramatic change in localization was observed, from diffusely cytoplasmic to a nuclear pattern with the overlapping signal of nuclear staining. Similar nuclear localization of ZfpA-GFP was observed under the stimulation of other azole drugs, such as fluconazole, voriconazole, and bifonazole. However, ZfpA-GFP maintained the cytoplasmic localization under the stimulation of terbinafine, calcium, caspofungin, calcofluor white, and Congo red (Fig. S3). Together, these results suggest that azole stresses induce the nuclear localization of ZfpA and further activate its expression in A. fumigatus.

### Loss of function of ZfpA influenced the response of A. fumigatus to itraconazole.

**FIG 4:**
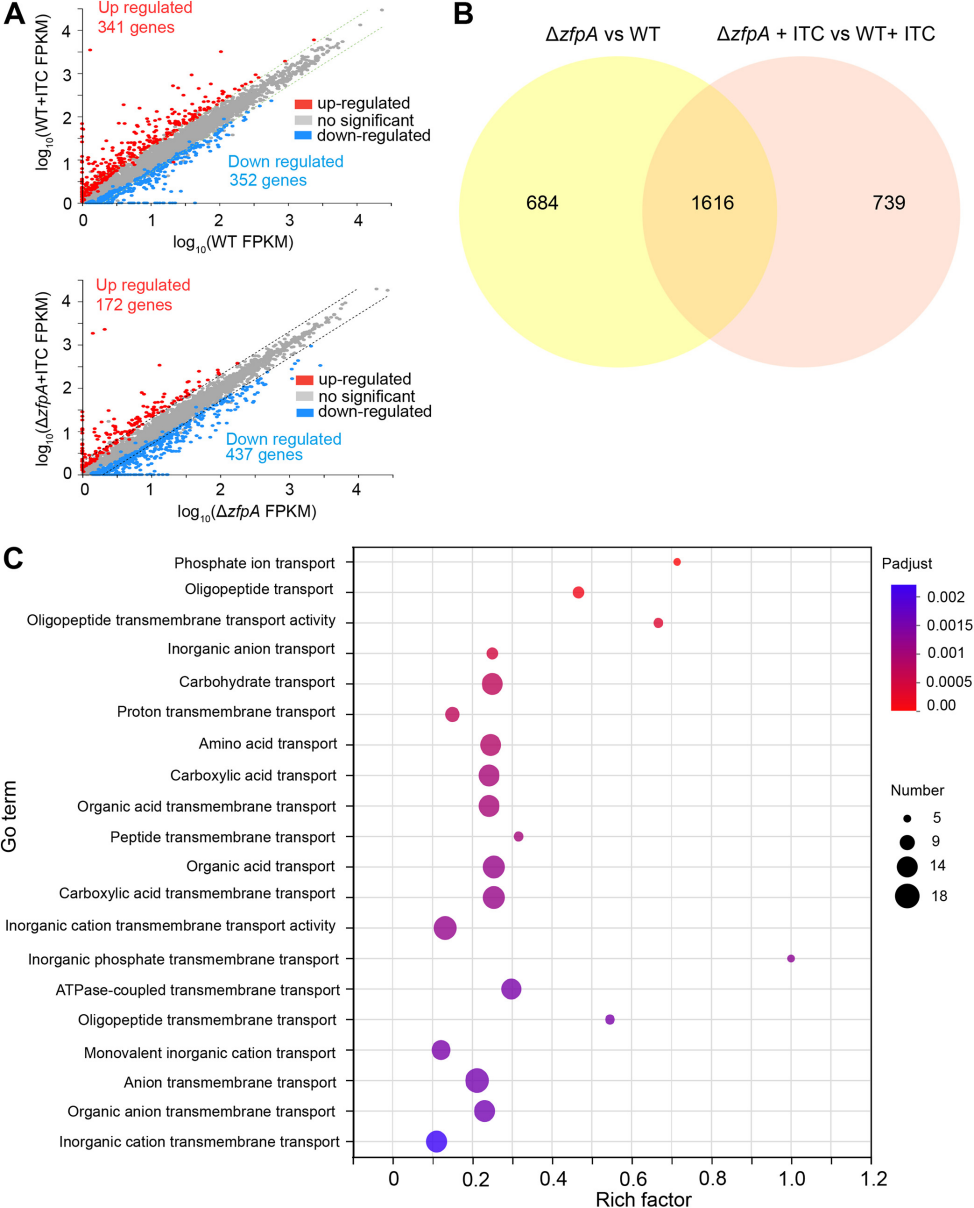
RNA-seq analysis of the whole-genome transcript profiles in the *zfpA* deletion strain compared to that of the parental wild-type strain with or without treatment of azole stress. (A) Volcano plot showing statistical significance (−log_10_ *P* value) versus fold change (log_2_ fold change) of RNA-seq data from wild-type and Δ*zfpA* mutant with or without ITC stimulus. Genes with increased expression (fold change ≥ 2 and *P* < 0.05) are shown in red, and genes with decreased expression (fold change ≤ 2 and *P* < 0.05) are shown in blue. (B) Venn diagram showing the overlapping genes in the *zfpA* deletion strain compared to that of the parental wild-type strain with or without azole stress. (C) Gene ontology analysis of 87 differentially expressed transport genes.

To decipher the role of ZfpA in the global regulation of gene expression in A. fumigatus, the wild-type and *zfpA* deletion transcriptomes were assessed postexposure to itraconazole. Differentially expressed genes were defined as those with a minimum of 2-fold change in gene expression and a false-discovery rate of less than 0.01. As shown in Fig. 4A, under the treatment of itraconazole, in the wild-type strain, 341 genes were upregulated and 352 genes were downregulated; in comparison, 172 genes were upregulated and 437 genes were downregulated in the *zfpA* deletion strain. A direct comparison of the two data sets revealed that the number of upregulated genes induced by itraconazole was decreased in the *zfpA* deletion strain compared to that in the wild-type strain, implying that loss of ZfpA influences the upregulation of genes response to itraconazole in A. fumigatus.

To further decipher changed genes induced by the deletion of *zfpA* which may contribute to drug susceptibility, we compared the *zfpA* mutant versus the wild-type strain with and without treatment of itraconazole, respectively. Venn diagram analysis revealed that the changed expression of 1,616 overlapping genes resulted from the loss of *zfpA* no matter whether it was with or without treatment of itraconazole (Fig. 4B), suggesting that ZfpA is required for the normal expression of these subsets of genes. Notably, gene ontology (GO) terms in the cluster analysis revealed that the loss of ZfpA significantly influenced the genes involved in catalytic activity and transporter activity (Fig. S4A). After analysis of different transporters, we found some ATPase-coupled transmembrane transporters which are involved in drug resistance (Fig. 4C). Of the ABC transport genes that showed the greatest increase in expression upon loss of *zfpA*, *atrF*, *atrB*, *mdr4*, *mfsA*, and *ste6* appeared (Table 2). Thus, these data indicate that the loss of ZfpA is capable of inducing the high expression of drug efflux pump-encoding genes, which had been demonstrated for having a relationship with drug susceptibility, implying that ZfpA is a negative regulator for drug resistance.

**TABLE 2:** Selected ABC transporter genes that may contribute to drug resistance in *zfpA* deletion strain

Gene name (A. fumigatus/S. cerevisiae)	Gene ID	Description	Fold changes Δ*zfpA*/WT (log_2_)	
			0	+ITC
*mdr4*/*ste6*	AFUB_012160	ABC multidrug transporter Mdr4	6.11	7.54
*ybt1*/*ybt1*	AFUB_036960	ABC transporter	5.64	5.03
*atrF*/*snq2*	AFUB_093930	ABC drug exporter AtrF	4.00	4.15
*atrB*/*snq2*	AFUB_047000	ABC multidrug transporter, putative	3.96	3.87
*abcA*/*pdr15*	AFUB_030790	ABC multidrug transporter, putative	1.73	2.08
*abc4*/*ybt1*	AFUB_066250	ABC multidrug transporter, putative	1.72	1.73
*ste6*/*ste6*	AFUB_045530	ABC multidrug transporter, putative	1.21	1.11
*mdr1*/*ste6*	AFUB_053630	ABC multidrug transporter Mdr1	1.01	2.47
*abcB*/*ycf1*	AFUB_048790	ABC multidrug transporter, putative	−2.07	−2.07

### Lack of ZfpA induces the upregulation of a series of ABC transport genes, and overexpressed *atrF* contributes to decreased azole susceptibility.

**FIG 5:**
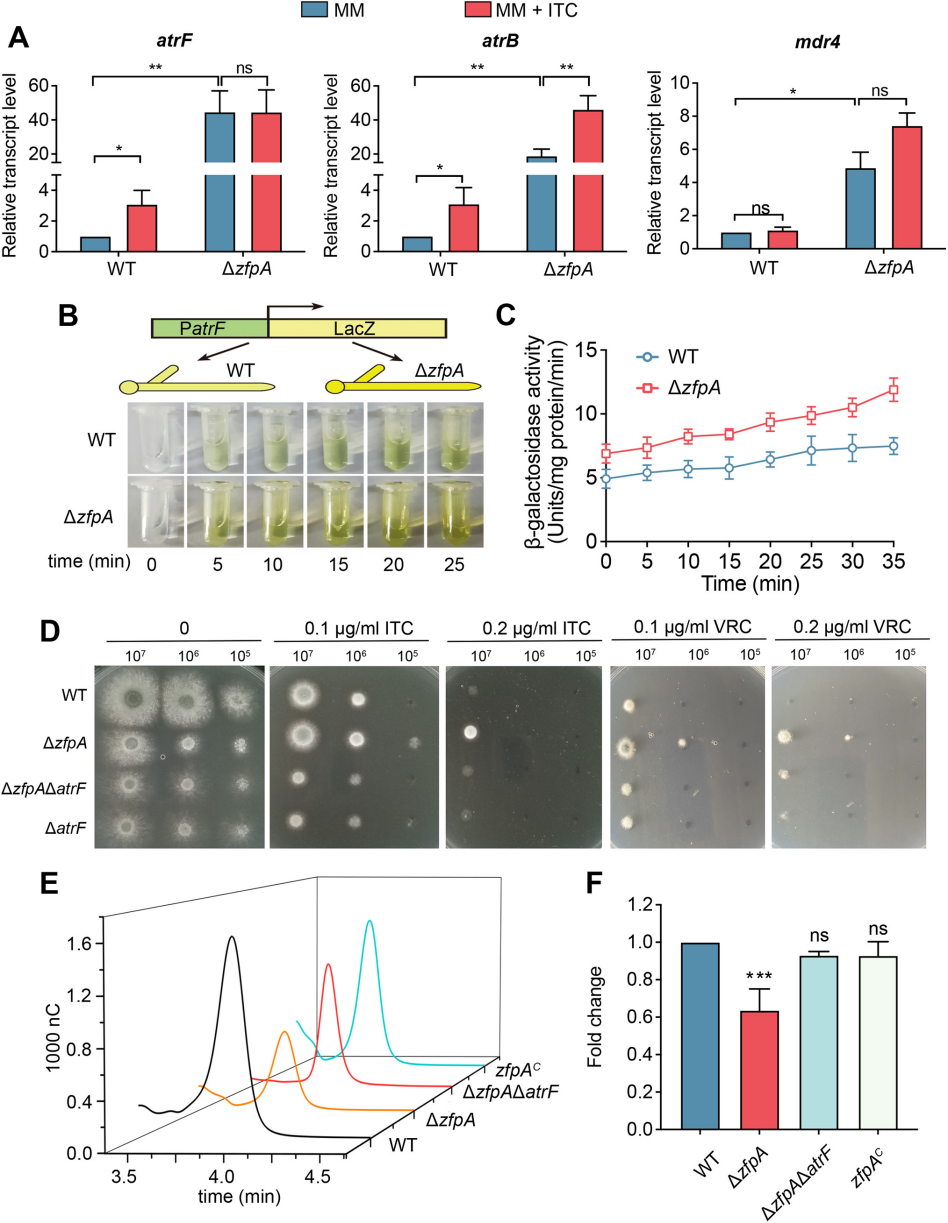
The loss of ZfpA induces the upregulation of a series of ABC transport genes. (A) The upregulation of the indicated ABC transport genes was verified by qRT-PCR. (B and C) The transcript level of the multidrug transport gene *atrF* was confirmed by β-galactosidase activity assay. (D) The indicated strains were inoculated onto the solid medium at 37°C for 2 days (without drugs) or 3 days (with drugs). (E and F) The intracellular ITC concentrations in the indicated strains were analyzed by HPLC. mAU, milliabsorbance units. Normalized quantification and comparison of the ITC contents in mutants and the parental wild-type strain. Statistical significance was determined using a 2-tailed *t* test. ns, not significant; *, *P < *0.05; **, *P < *0.01; ***, *P < *0.001.

In order to verify whether the decreased azole susceptibility in the *zfpA*-null mutant resulted from the overexpression of ABC transporter genes, quantitative real-time PCR (qRT-PCR) in additional biological replicate samples was performed to confirm the differential expression of these genes. Notably, the putative ABC transport genes *atrF*, *atrB*, *mdr4*, *ste6*, *ybt1*, and *mdr1* showed remarkable upregulation in the *zfpA*-null mutant compared to the parental wild-type strain under both the normal condition and the ITC-stimulated condition (Fig. 5A and Fig. S4B), suggesting that the majority of these selected gene expression differences were consistent with those in the transcriptome sequencing (RNA-seq) data set. Interestingly, compared to its parental wild-type strain, the *zfpA*-null mutant displayed maximal mRNA changes in the ABC transport gene *atrF* (approximately 40-fold increase), which is a key drug resistance-related gene by actively extruding drugs out of the intracellular environment (10), among all ABC transport genes. We further visualized the differential expression between the mutant and parental wild-type strain by fusing the *atrF* promoter with a bacterial *lacZ* reporter gene. As shown in Fig. 5B and C, the β-galactosidase activity with a yellow color was increased in samples extracted from the *zfpA*-null mutant compared with that of the parental wild-type strain, indicating that the promoter of *atrF* was truly hyperactivated in the *zfpA*-null mutant. To further address whether *atrF* was indispensable for the altered azole susceptibility of the *zfpA*-null mutant, an *atrF* deletion was then constructed in both the parental wild-type and *zfpA*-null mutant backgrounds. As shown in Fig. 5D, the Δ*zfpA*Δ*atrF* double mutant showed increased azole susceptibility compared to the *zfpA* null mutant, suggesting that the decreased azole susceptibility of the *zfpA* deletion strain is dependent on the expression of *atrF*. Moreover, the high-performance liquid chromatography (HPLC) assay further identified that the deletion of *atrF* in the *zfpA*-null mutant significantly recovered the low cytosolic itraconazole contents toward a wild-type level (Fig. 5E and F). Together, these data suggested that the decreased azole susceptibility in the *zfpA* deletion strain results from the overexpression of the ABC transport gene *atrF* accompanied by reduced cytosolic ITC contents.

### Overexpression of *atrF* in the *zfpA* deletion strain is dependent on CrzA.

**FIG 6:**
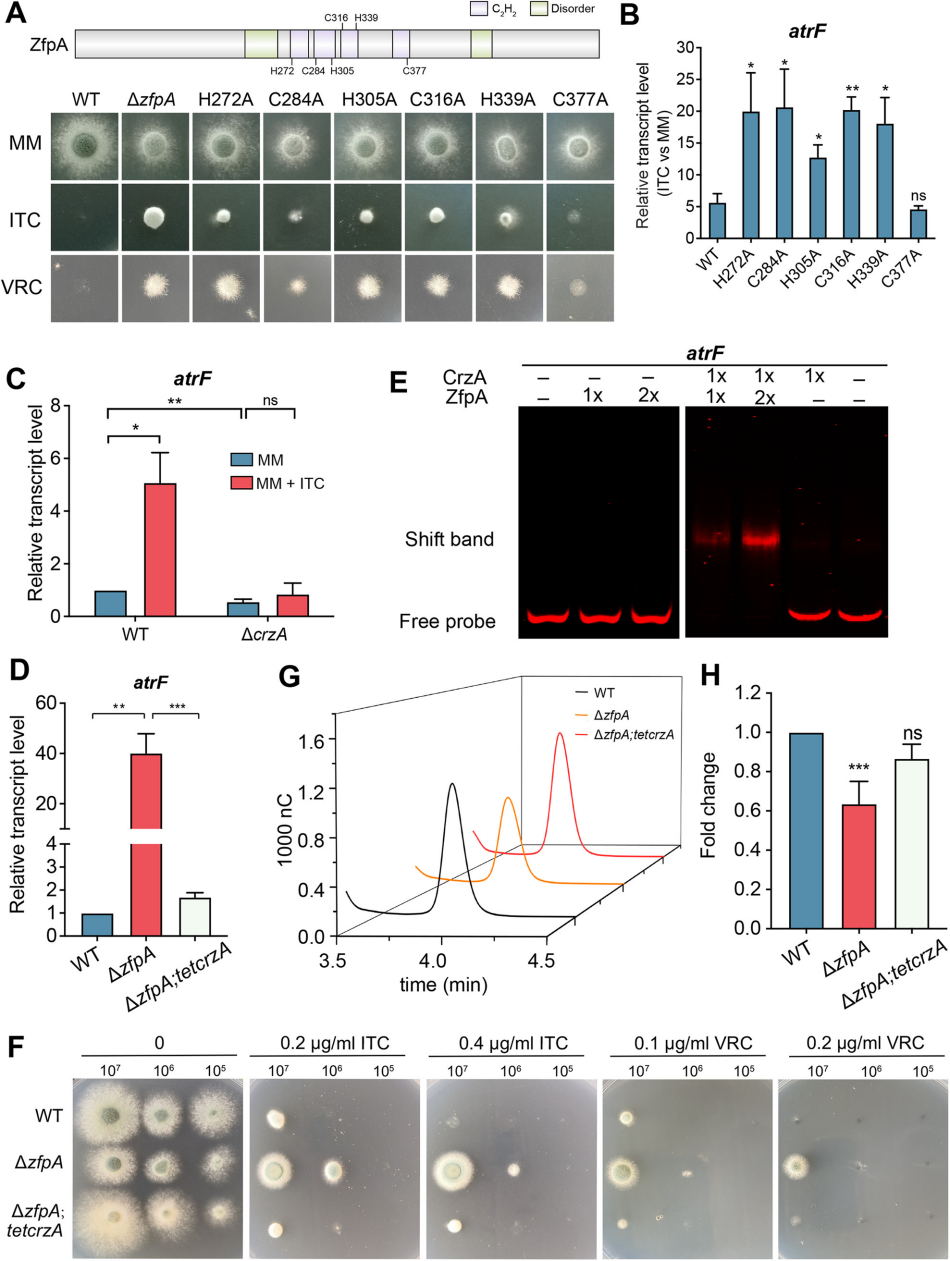
ZfpA cooperates with CrzA to regulate the expression of the multidrug transporter gene *atrF.* (A) Colony phenotypes of the indicated strains on the solid medium with ITC and VRC. (B to D) Expression changes of mRNA in the indicated strains identified by qRT-PCR for the multidrug transporter gene *atrF*. (E) EMSA showed the *in vitro* binding of CrzA and ZfpA to Cy5-labeled promoter fragment 2 of gene *atrF*. (F) The colony phenotype of the indicated strains on MM with various azole concentrations. (G and H) The intracellular ITC concentrations in the indicated strains were analyzed by HPLC. mAU, milliabsorbance units. Normalized quantification and comparison of the ITC contents in mutants and the parental wild-type strain. Statistical significance was determined using a 2-tailed *t* test. ns, not significant; *, *P < *0.05; **, *P < *0.01; ***, *P < *0.001.

Next, we sought to identify the regulatory mechanism by which ZfpA regulates the expression of *atrF* in A. fumigatus. According to the conserved motif analysis by aligning the selected homologs, they all had canonical zinc finger C_2_H_2_ regions (Fig. 6A). Based on this finding, we constructed site mutations by individually substituting cysteine (Cys, C) or histidine (His, H) with alanine (Ala, A) to assess the function of the conserved C_2_H_2_ domain of ZfpA (Fig. 6A). Notably, the conserved site mutations exhibited a similar growth defect to the *zfpA* deletion strain and similar azole susceptibility except for the C377A mutation (Fig. 6A and Fig. S5). To further verify whether the decreased azole susceptibility of these mutations was due to the overexpression of *atrF*, we tested the transcriptional level of *atrF* in *zfpA* mutants under azole stimulation. In the wild-type strain, azole stimulation caused a 5-fold-increased expression of *atrF* compared to that under normal conditions. Moreover, *zfpA* mutants all exhibited a higher increment than the wild-type strain except for the C377A mutation (Fig. 6B). These data suggested that these amino acid residues within the C_2_H_2_ domains of ZfpA are critical for azole susceptibility and regulating the expression of *atrF*. Because the C_2_H_2_ zinc finger domain is the most common DNA binding domain, we sought to examine whether ZfpA could directly bind to the promoter of *atrF*. Thus, we expressed ZfpA in Escherichia coli and analyzed protein-DNA interactions by EMSAs. However, we did not detect any interactions between ZfpA and the tested promoter region of *atrF in vitro* (Fig. S6A and B).

Notably, as shown in Fig. 6C, expression of *atrF* could be affected by *crzA* such that the *crzA* deletion mutant abolished enhanced expression of *atrF* in the treatment of azole compared to the parental wild-type strain. According to these results, we speculated that CrzA might be also required for the overexpression of *atrF* in the *zfpA* deletion strain. To address this hypothesis, we constructed a conditionally expressed CrzA strain under the control of the *Tet* promoter in the *zfpA* deletion strain in which the expression of *crzA* was repressed without doxycycline. As shown in Fig. 6D, the downregulated *crzA* in the *zfpA* deletion strain markedly decreased the expression of *atrF*, implying that the overexpression of *atrF* in *the zfpA* deletion strain is dependent on the normal expression of *crzA*. Since CrzA is required for the overexpression of *atrF* in the *zfpA* deletion strain and ZfpA cannot bind to the promoter of *atrF* by itself, we speculated whether CrzA is also important for ZfpA to bind to the promoter of *atrF*. To address this question, we performed the EMSA with the *atrF* promoter in the presence of both CrzA and ZfpA. Strikingly, we found that the promoter of *atrF* showed binding in the presence of both CrzA and ZfpA compared to that only with ZfpA (Fig. 6E and Fig. S6C). These data suggest that the expression of *atrF* regulated by ZfpA is dependent on the transcription factor CrzA. To further examine the drug susceptibility, we performed the phenotypic assay and found that the downregulated *crzA* in the *zfpA* deletion strain significantly increased the azole susceptibility compared to the *zfpA* deletion strain (Fig. 6F). Moreover, the upregulated *crzA* in the *zfpA* deletion strain obviously caused more azole tolerance than that in the *zfpA* deletion strain (Fig. S7A and B). Consistently, the HPLC assay identified that downregulation of *crzA* in the *zfpA*-defective strain significantly recovered the low cytosolic itraconazole contents toward a wild-type level (Fig. 6G and H). Together, these data suggested that the decreased azole susceptibility caused by overexpression of *atrF* in the *zfpA* deletion strain is dependent on the expression of *crzA*.

## DISCUSSION

The transcriptional network governing azole resistance is highly complex and involves multiple regulators that remain to be fully identified in fungal pathogens (1). The well-studied Pdr1 and its paralog, Pdr3, are zinc finger transcription factors that regulate the pleiotropic drug response in S. cerevisiae. Pdr1 and Pdr3 serve as transcriptional activators and repressors by binding to pleiotropic drug response elements (PDREs) in the promoter regions of ABC transporter genes, including *pdr5*, *pdr10*, and *pdr15* (28). In Candida albicans, the transcription factors Tac1, Mrr1, Upc2, and Yap1 have been reported to regulate multidrug transporter genes linked to azole resistance (12). Although the number of key regulators regulating drug resistance in mold and pathogenic yeasts have been uncovered, the roles of transcription factors can vary significantly from species to species (28). In recent years, there have been reports on the important roles of various A. fumigatus transcriptional regulators in response to antifungal drugs (29–31). The sterol regulatory element binding protein SrbA is a transcriptional activator that directly regulates at least seven genes in the ergosterol biosynthetic pathway, including *cyp51A* (31). In addition, AtrR, a Zn2-Cys6 transcription factor, also positively regulates sterol biosynthesis and directly binds the promoter of the *cyp51A* and *cdr1B* genes (29). The CCAAT binding domain complex CBC, a heterotrimer comprising HapB, HapC, and HapE, is a negative regulator of sterol biosynthesis that directly binds the promoters of 14 ergosterol biosynthetic genes, including *cyp51A* (32). NctA and NctB, CBF/NF-Y family transcription regulators, are key regulators of ergosterol biosynthesis and the drug exporter Cdr1B (30). However, there are still many transcription factors and regulators that remain to be identified to explain the mechanism of azole resistance.

AtrF, as an ABC transporter, was identified from one itraconazole-resistant clinical isolate which accumulates low levels of itraconazole due to *atrF* overexpression (10). Our previous work found that the overexpression of *atrF* is dependent on the expression of *crzA* (22); however, *in vitro* EMSA showed that CrzA does not bind the promoter of *atrF*. Thus, we reanalyzed the published ChIP-seq data of CrzA and identified that the expression of the transporter gene *atrF* was regulated by ZfpA, which was previously found to be involved in oxylipin response and drug resistance (17, 24, 25). Our RNA-seq analysis showed that several transmembrane transporters were differentially expressed, and the expression level of *atrF* was dramatically increased in the *zfpA*-null mutant strain. Moreover, the decreased azole susceptibility of the *zfpA* deletion strain is able to be rescued by overexpressed *atrF*, suggesting that *atrF*-encoded ABC transport might be a major contributor to the *zfpA*-involved azole susceptibility. Notably, findings in this study regarding the decreased azole susceptibility in *zfpA*-null mutant are consistent with the previous report that *zfpA* deletion shows an increased MIC of itraconazole in the CEA17 background strain (24). However, it is different from the recent study in which it was found that the *zfpA* deletion strain showed slightly higher susceptibility to voriconazole in the CEA10 background strain (26). We speculate that there are two possibilities that led to the inconsistent conclusion regarding the susceptibility of the *zfpA* deletion strain. First, there may be variations in the strain background used in the study. Second, differences in the culture conditions for conidial preparation, variations in inoculation methods, and different sources of voriconazole may also contribute to slightly different results (33). Besides the azole tolerance, the deletion of *zfpA* also influenced the susceptibility of the allylamine antifungal terbinafine (Fig. 2C) and echinocandin antifungal caspofungin (24–26). Among the differentially expressed ABC transporter genes in the *zfpA* deletion strain, it is possible that some ABC transporter genes contribute to the alteration of drug susceptibility in the *zfpA* deletion strain.

The amino acid residues within the C_2_H_2_ domains in ZfpA were critical for repressing the expression of *atrF* (Fig. 6A and B). However, we did not detect any interactions between ZfpA and the tested promoter region of *atrF in vitro*. Most interestingly, the repressed expression of *atrF* by ZfpA is dependent on CrzA. Only CrzA or ZfpA cannot bind to the promoter of *atrF*, while ZfpA showed the binding of the *atrF* promoter in the presence of CrzA (Fig. 6E and Fig. S6C), suggesting that CrzA and ZfpA may form a complex to bind the promoter of *atrF* and regulate its expression. Although ZfpA was identified to be a putative targeted protein by CrzA in its ChIP-seq data, we found that *crzA* deletion did not influence the expression of *zfpA* (Fig. S7C), suggesting that ZfpA may not be the downstream target of CrzA. Moreover, the deletion of *zfpA* did not influence the expression of *crzA*, and ZfpA cannot bind to the promoter of *crzA* (Fig. S7D). Thus, the relationship between CrzA and ZfpA is still unclear. Based on the bioinformatic analysis, we found that ZfpA contains two intrinsically disordered regions which have been shown to contribute to liquid-liquid phase separation (34). Whether ZfpA interacts with CrzA through phase separation needs to be explored in future work.

ZfpA as a transcription factor not only regulates the genes involved in drug susceptibility but also influences the expression of genes in the metabolism pathway (Fig. S8). We found that the expression of genes involved in the tricarboxylic acid (TCA) cycle was decreased in both deletion and overexpression of *zfpA*. This finding indicated that ZfpA is important for the growth of A. fumigatus. However, the growth defects in the *zfpA* deletion and the overexpressed strains are more obvious in this study than in those previously reported (25, 26). As predicted, there are consistent data regarding the *zfpA*-involved animal virulence and hypersensitive colony phenotypes to the cell wall destruction reagent in both previous and our studies (26). Interestingly, the hyperbranching phenotypes caused by overexpressed *zfpA* were accompanied by altered cell wall chitin content in the previous work may suggest why and how the abnormal expression of *zfpA* affects animal virulence since hyphal branching in Aspergillus is important for acquiring nutrients and the host penetration (35–37).

**FIG 7:**
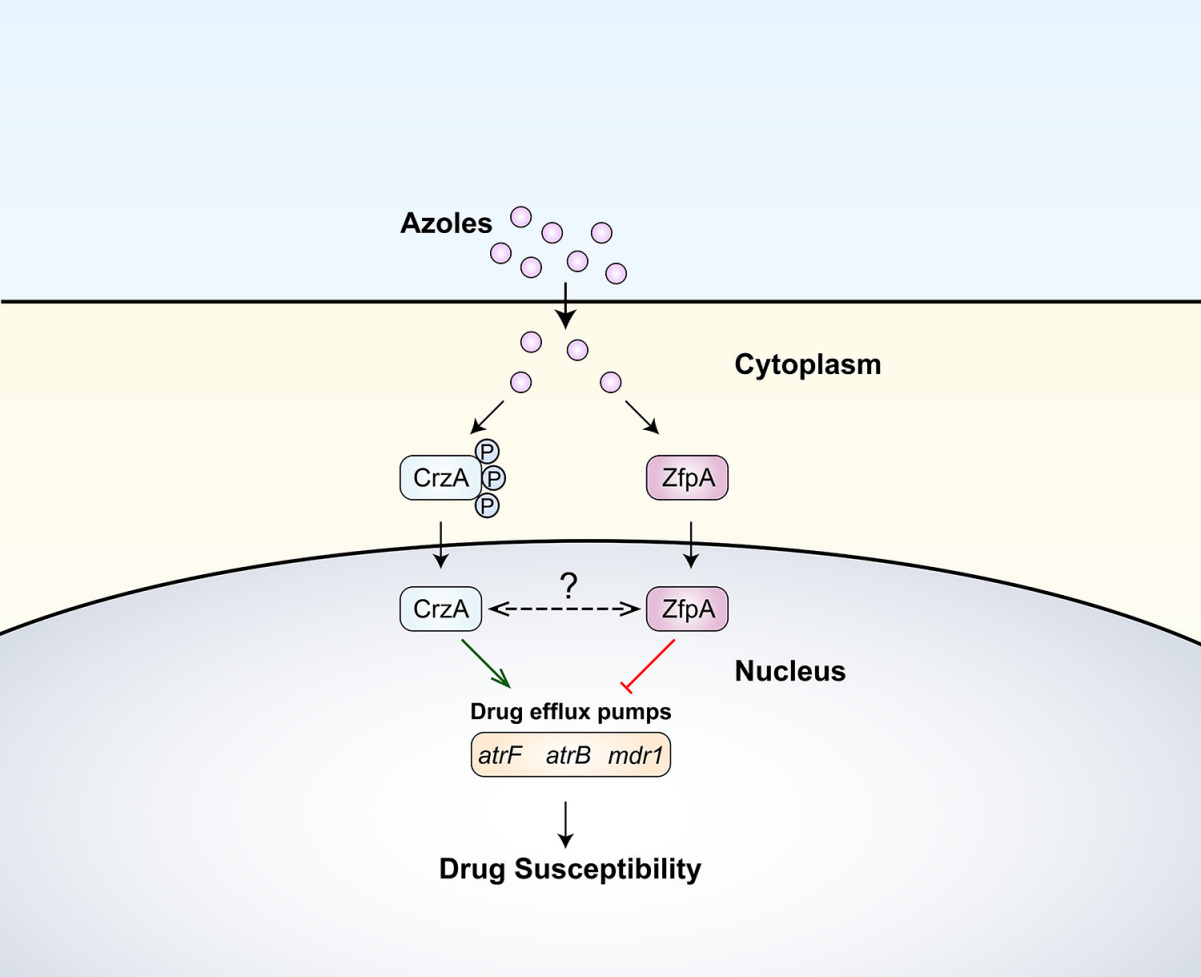
Schematic model showing how the transcription factors ZfpA and CrzA regulate the expression of drug efflux pump-encoding genes contributing to drug susceptibility. The positive regulator CrzA is activated by azole stresses and then translocated to the nucleus to induce the expression of drug efflux pump-encoding genes against antifungals. Alternatively, the negative regulator ZfpA is activated simultaneously and translocated to the nucleus to repress the overexpression of drug efflux pump-encoding genes. The green arrow represents the activation of transcription. The red line represents the repression of transcription. The double-orientated and dashed arrow line represents the unclear relationship between CrzA and ZfpA.

Taken together, our work suggests that CrzA, as a positive regulator, is activated by azole stresses and then translocated to the nucleus to evoke the expression of multidrug transporter genes against azole stresses, while ZfpA, as a negative regulator, is also activated and represses the overexpression of multidrug transporter genes, which is a homeostasis mechanism to restrain excessive expression levels of transporter genes (Fig. 7). These findings shed new light on how filamentous fungi react and adapt to azole stresses via the regulation of multidrug transporter genes.

## MATERIALS AND METHODS

### Strains, media, and cultural conditions.

The A. fumigatus strains used in this study are summarized in Table S1 in the supplemental material. The minimal medium (MM) included 1% glucose, 1 mL/L 1,000 × trace elements, and 50 mL/L 20 × salt solution (pH 6.5) (38). For all related liquid media, agars were omitted. The transformation protocol was as described in the references (39). All strains were incubated at 37°C for the indicated times.

### Deletion, overexpression, complementation, and site-directed mutation of *zfpA*.

All primers are shown in Table S2. To generate the *zfpA* deletion, the fusion PCR technique was used as described previously (40, 41). Briefly, approximately 1 kb of the upstream and downstream flanking sequences of the *zfpA* gene was amplified using the primer pairs ZfpA P1/P3 and ZfpA P4/P6, respectively. The selection marker gene *pyr4* was amplified from plasmid pAL5 with the primers pyr4 F/R. After the three aforementioned PCR products were obtained, the mixture was used as a template to generate the *zfpA* deletion cassette using the primer pair ZfpA P2/P5. This deletion cassette fragment was then used to transform the recipient strain A1160. To generate overexpression strains, the *zfpA* genes were amplified and subcloned into the ClaI site of the pBARGPE-1 vector containing the constitutive *AngpdA* promoter. Subsequently, the overexpressed plasmid was integrated into the genome of the parental strain A1160. To reconstitute Δ*zfpA* with a functional copy of the *zfpA* gene, the following strategy was used. First, the fragment of *zfpA,* including the native promoter, 5′ untranslated region (5′ UTR), gene sequence, and 3′ UTR, was amplified with the primer pair ZfpA-XbaI-F/ZfpA-HindIII-R and then subcloned into the pAN7-1 vector. The plasmids were then used to transform the Δ*zfpA* strain. For site-directed mutagenesis, primers harboring the desired mutation were synthesized and then used for PCR using the complemented plasmid as a template. The plasmid harboring the indicated mutations was then transformed into the *zfpA*-null strain. All transformants were confirmed by diagnostic PCR, and mutations in transformants were identified by sequencing.

### G. mellonella virulence assay.

Experiments in G. mellonella were performed as described previously (42). Briefly, 10 μL (8 × 10^7^ conidia/mL) from the indicated strains was infected in G. mellonella, and 10 μL of phosphate-buffered saline (PBS) served as the control. Then, the larvae were incubated at 37°C in the dark and monitored daily (15 days). The survival rate was evaluated using Kaplan-Meier survival curves and analyzed with the log-rank (Mantel-Cox) test utilizing GraphPad Prism software. Differences were considered significant at *P* values of <0.05.

### Fluorescence microscopic analyses.

To visualize the localization of ZfpA, the strains were grown on coverslips in 1 mL liquid MM at 37°C for 12 h. Itraconazole was added to a final concentration of 4 μg/mL and incubated for 1 h. Cultured cells were then fixed with 4% paraformaldehyde and washed three times with PBS. Then, the nuclear dye 4′,6-diamidino-2-phenylindole (DAPI) dissolved in PBS was used at a final concentration of 1 μg/mL and incubated for 30 min at room temperature. The DAPI solution was then removed, and the glass coverslip was washed three times with PBS. Images were captured using a Zeiss Axio Imager A1 microscope (Zeiss, Jena, Germany) and organized by Adobe Photoshop software.

### Total intracellular drug detection.

We inoculated 10^7^ conidia into 100 mL liquid MM, and they were shaken on a rotary shaker at 220 rpm at 37°C for 24 h. Itraconazole was added at a final concentration 4 μg/mL. After incubation for 1 h, the mycelia were washed, harvested, and lyophilized. Cellular itraconazole was extracted by dry mycelia and analyzed as previously described (40).

### RNA extraction for qRT-PCR.

We inoculated 10^7^ conidia into 100 mL liquid MM, and they were shaken on a rotary shaker at 220 rpm at 37°C for 24 h. Subsequently, the collected mycelia were frozen with liquid nitrogen. For qRT-PCR analysis, total RNA samples were extracted and purified from the frozen mycelium using TRIzol (Roche) as described in the manufacturer’s manual. The digestion of genomic DNA and synthesis of cDNA were performed according to the protocol described in the HiScript II Q RT supermix for qPCR (+gDNA wiper) kit (Vazyme).

### β-Galactosidase activity assay.

A. fumigatus conidia were inoculated into liquid MM media and shaken on a rotary shaker at 220 rpm at 37°C for 24 h. Subsequently, the collected mycelia were frozen with liquid nitrogen. β-Galactosidase assays were performed as previously described (22). Samples were analyzed from three independent biological replicates.

### Recombinant ZfpA protein purification and electrophoretic mobility shift assay.

The pGEX-AfZfpA harboring the A. fumigatus *zfpA* full-length cDNA was transformed into E. coli BL21. Then, the glutathione *S*-transferase (GST) fusion protein was purified following the manual of the relative products. For the EMSA probe, Cy5-labeled probes were prepared as described in the references. The other EMSA procedure was performed as described previously (43, 44).

### Antifungal susceptibility testing.

Etest assay was performed in accordance with the manufacturer's instructions as described previously (45). The agar formulation used for the Etest was RPMI 1640 supplemented with 1.5% agar and 2% glucose and buffered to pH 7.0 with morpholinepropanesulfonic acid (MOPS) buffer. A 2 × 10^7^ spore suspension was plating and streaking it across the surface of the agar in three directions. The plates were dried at ambient temperature for 15 min before applying voriconazole and itraconazole Etest strips (catalog no. S21Z07 and S21Z03; Bio-Kont, China). The plates were incubated at 37°C and read at 48 h.

### Data availability.

For RNA-seq analysis, all samples were sent out for digital transcriptome analyses by a standard RNA-seq approach in Shanghai Majorbio Bio-pharm Technology Co., Ltd. The raw Illumina sequencing data have been submitted to SRA (https://www.ncbi.nlm.nih.gov/sra) at NCBI with accession numbers SRR23886333 to SRR23886338.
