# Supplementary material for: The C2H2-Type Transcription Factor ZfpA, Coordinately with CrzA, Affects Azole Susceptibility by Regulating the Multidrug Transporter Gene atrF in Aspergillus fumigatus
Source: Microbiol Spectr. 2023 Jun 15;11(4):e00325-23. doi: 10.1128/spectrum.00325-23 (PMC10434176; doi:10.1128/spectrum.00325-23)
Supplement: Supplemental file 2 — Fig. S1. Download spectrum.00325-23-s0002.docx, DOCX file, 5.6 MB [file spectrum.00325-23-s0002.docx]

**The C_2_H_2_-type transcription factor ZfpA coordinately with CrzA affects azole susceptibility by regulating the multidrug transporter gene *atrF* in *Aspergillus fumigatus***

Yeqi Li, Mengyao Dai, *Ling Lu, *Yuanwei Zhang

Jiangsu Key Laboratory for Microbes and Functional Genomics, Jiangsu Engineering and Technology Research Centre for Microbiology, College of Life Sciences, Nanjing Normal University, Nanjing, China

Running title: Negative regulator ZfpA of multidrug transporter gene *atrF*

Key words: drug susceptibility, negative regulator, ABC transporter, fungal pathogen, *Aspergillus fumigatus*

Yeqi Li and Mengyao Dai contributed equally to this work.

Address correspondence to Ling Lu, linglu@njnu.edu.cn and Yuanwei Zhang, [ywzhang@njnu.edu.cn](mailto:ywzhang@njnu.edu.cn)


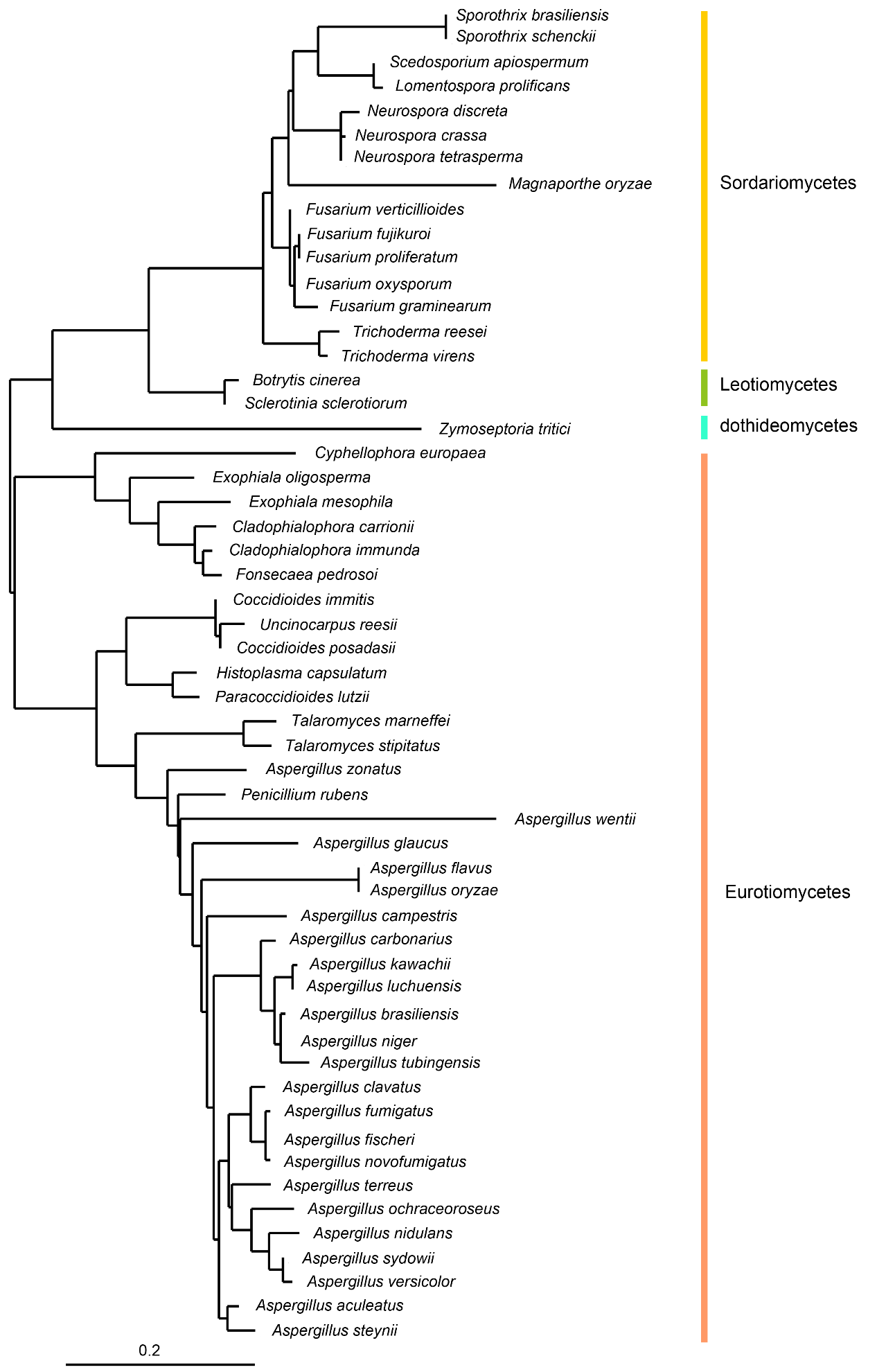


Fig. S1 Bioinformatics analysis of ZfpA.

The BLASTP analysis in all fungi by using ZfpA protein sequence found all potential homologs. The Phylogenetic tree was constructed and analysis of ZfpA homologs from all species by using MEGA 5 software.


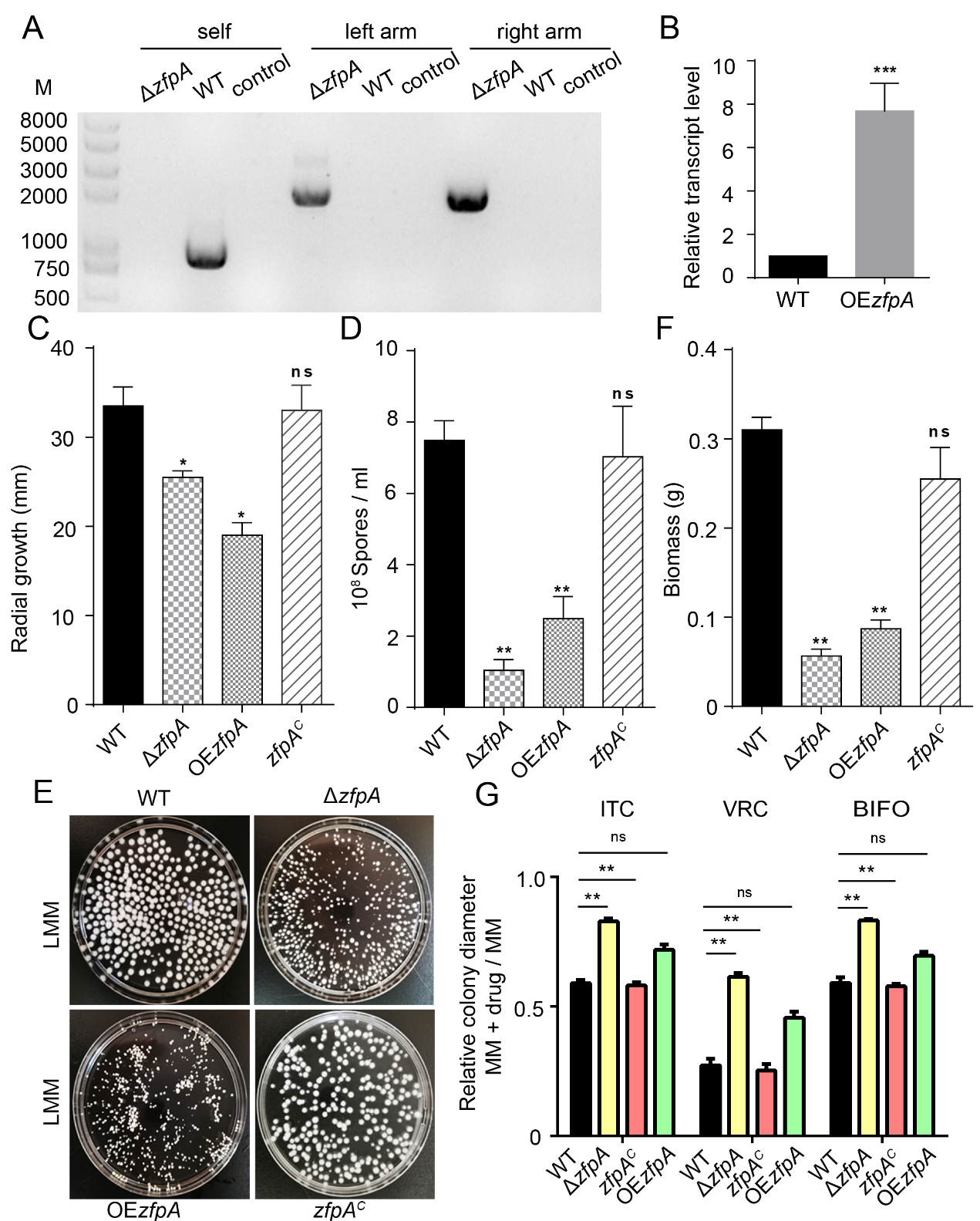
Fig. S2 The construction and quantification for the indicated strains.

(A) The diagnostic PCR of the deletion strains compared to the wild-type strain. (B) qRT-PCR verified the expression of *zfpA* in the indicated strain. (C-D) The indicated strains were cultured in the solid minimal medium or liquid minimal medium at 37 °C for 2 days. The colony diameter in the solid medium was measured (C) and then collected the spores for quantification by using the hemocytometer (D). (E-F) The indicated strains were cultured in the liquid minimal medium at 37 °C with 220 rpm for 2 days, and then collected (E), lyophilized for biomass quantification (F). (G) The relative colony diameter in the different drug conditions. Statistical significance was determined using a 2-tailed t test. ns, not significant; *, *P* < 0.05; **, *P* < 0.01; ***, *P* < 0.001.


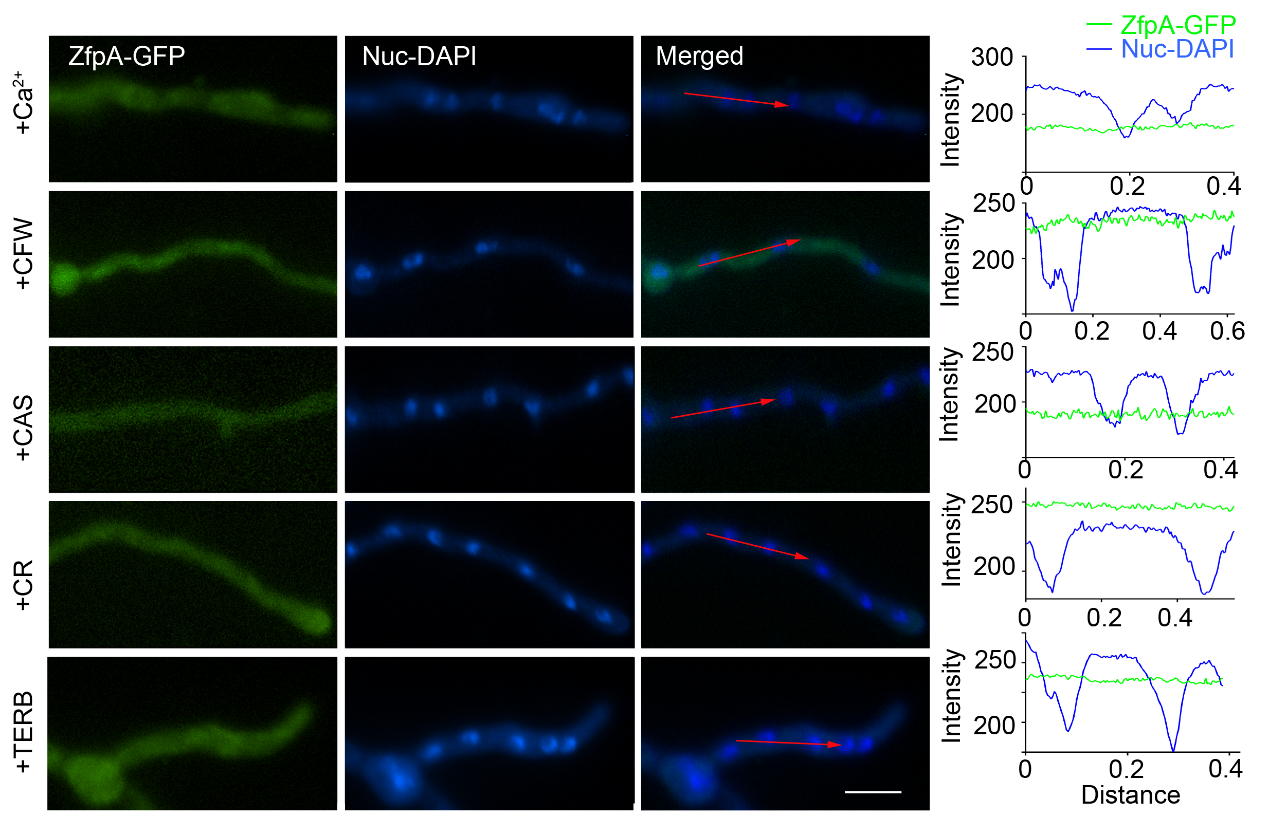


Fig. S3 Subcellular localization of ZfpA in *A. fumigatus*

Epifluorescence microscopic images demonstrating the ZfpA-GFP distribution under the untreated or treated conditions with Ca^2+^ (100 mM), CFW (40 μg/ml), CAS (1.25 μg/ml), CR (30 μg/ml) and TERB (0.5 μg/ml) for 30 min. DAPI was a nuclear localization signal dye used to visualize the nucleus. The merged images of GFP and DAPI stainings showed nuclear localization of ZfpA-GFP. The green line represented ZfpA-GFP, and the blue line represented DAPI. Distribution patterns of ZfpA-GFP and DAPI analyzed by the software Image J. Bars, 10 μm.


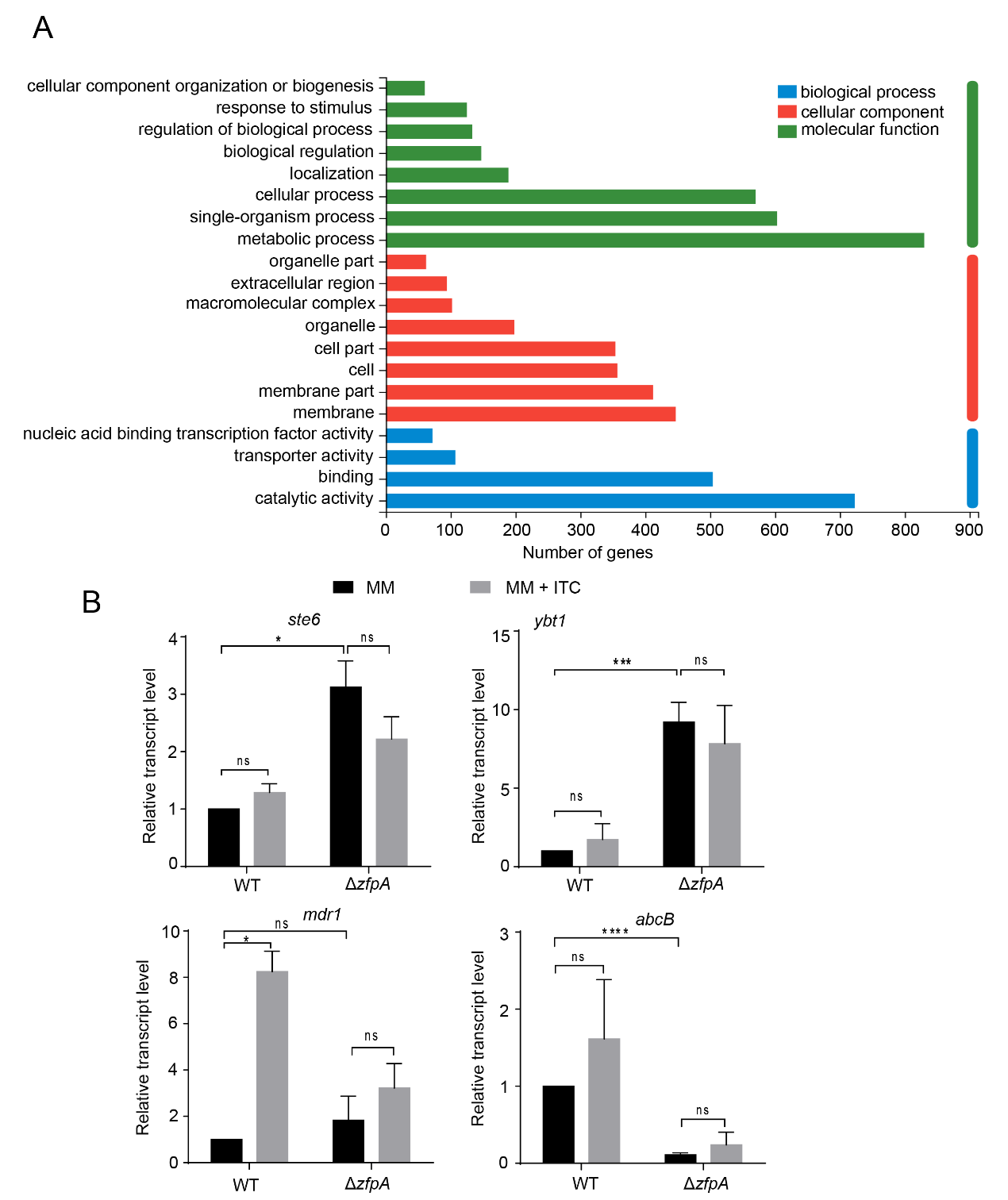


Fig. S4 Gene Ontology analysis of the overlapping genes and expression changes of mRNA identified by qRT-PCR for multidrug transporter genes.

(A) Gene Ontology analysis of the 1616 overlapping genes influenced by the deletion of *zfpA*. (B) The expression of multidrug transporter genes was identified by qRT-PCR. Statistical significance was determined using a 2-tailed t test. ns, not significant; *, *P* < 0.05; ***, *P* < 0.001.


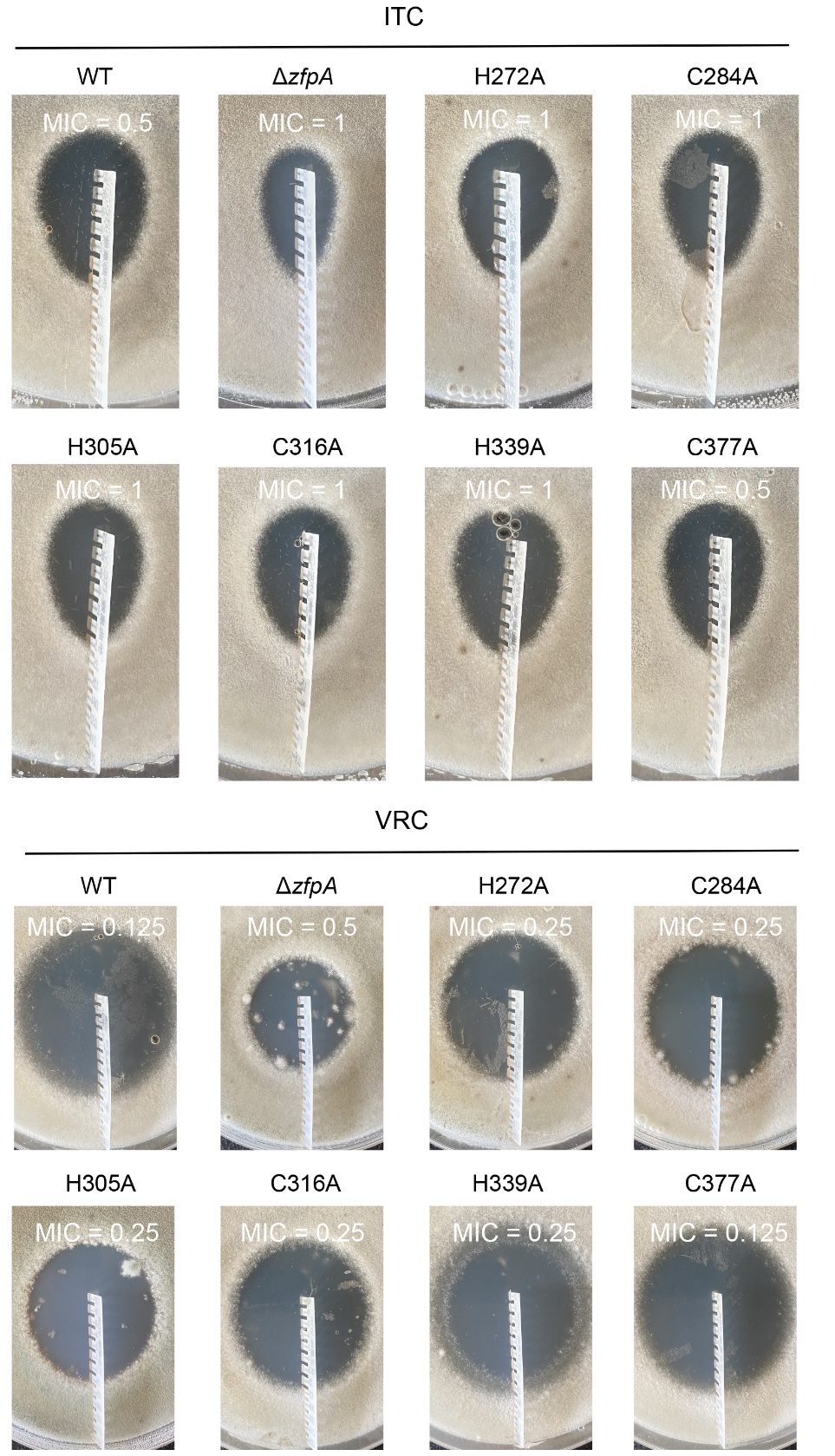


Fig. S5 Susceptibility of *A. fumigatus* wild type and *zfpA* mutant strains.

RMPI agar plates were inoculated with 2 x 10^7^ conidia of the indicated strains. Etest strips for itraconazole (ITC) and voriconazole (VRC) were applied, and plates were incubated at 37 °C for 2 days.


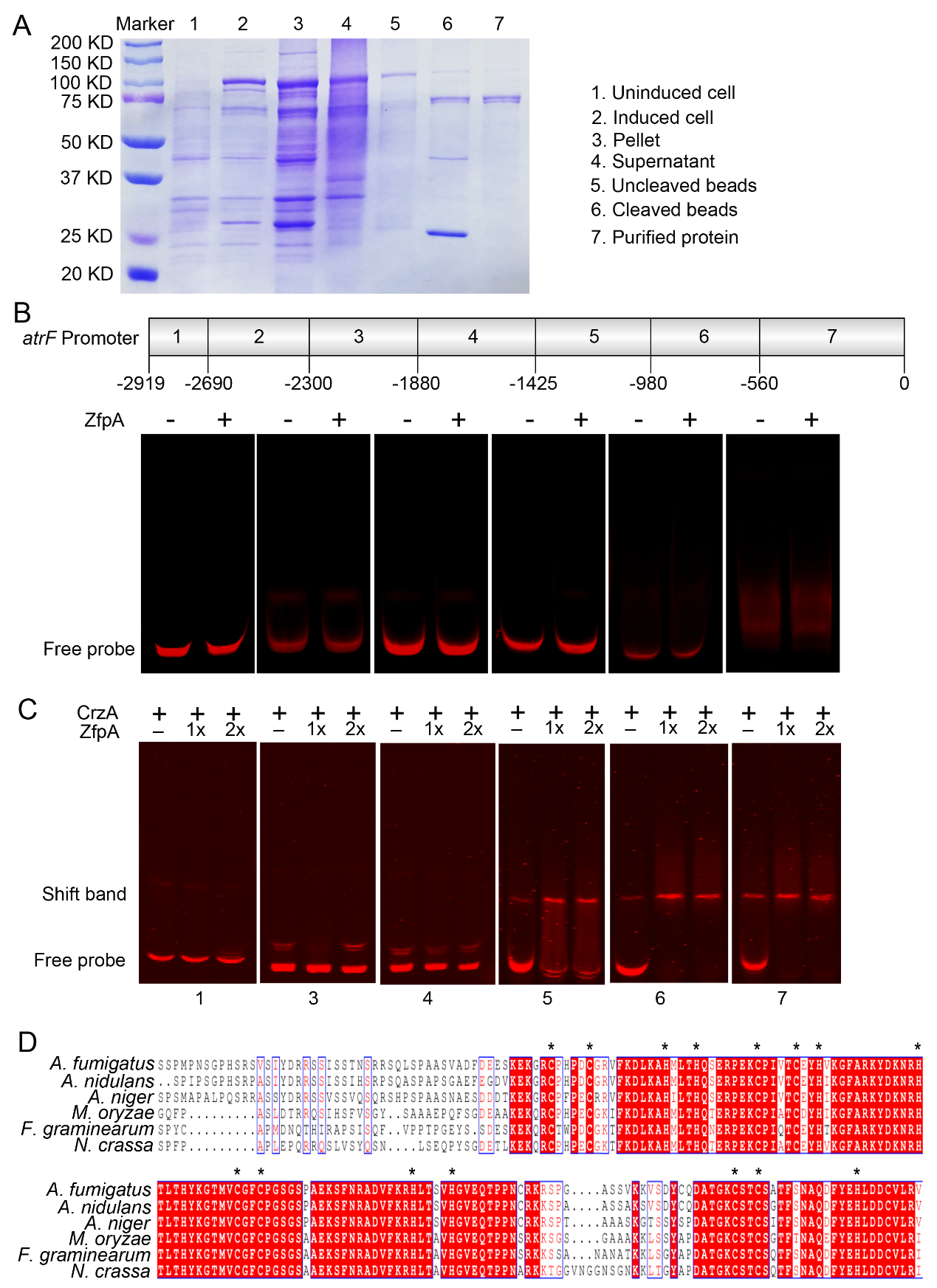


Fig. S6 EMSA of the ZfpA protein for *atrF* promoter and conserved sites analysis for ZfpA.

(A) SDS-PAGE analysis of the recombinant ZfpA produced in *E. coli*. (B) EMSA analysis of ZfpA protein and Cy5-labeled promoter fragments of *atrF*. (C) EMSA analysis of Cy5-labeled promoter fragments of *atrF* in the presence of both ZfpA and CrzA. (D) Alignment of ZfpA homologs and analysis of conserved amino acid residues in selected fungi.


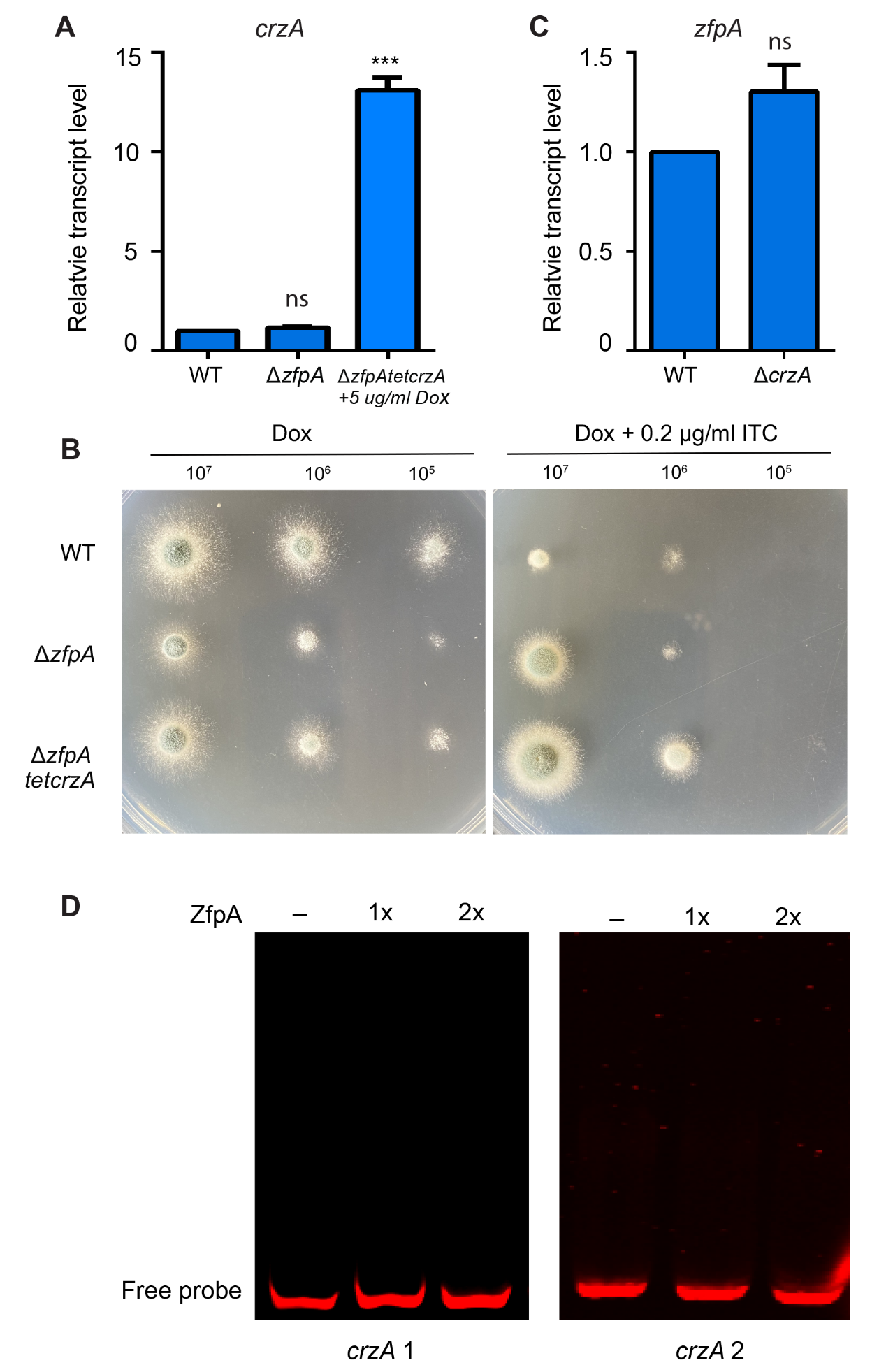


Fig. S7 Overexpressed *crzA* in the *zfpA* deletion affects the drug susceptibility and EMSA of the ZfpA protein for *crzA* promoter.

(A) The indicated strains were grown in liquid MM in the presence of 5 μg/ml doxycycline. The expression of *crzA* was identified by qRT-PCR. (B) The indicated strains were cultured in the solid MM in the presence of 5 μg/ml doxycycline with or without 0.2 μg/ml itraconazole. (C) The expression of *zfpA* in the indicated strains was identified by qRT-PCR. (D) EMSA analysis of ZfpA protein and Cy5-labeled promoter fragments of *crzA*.


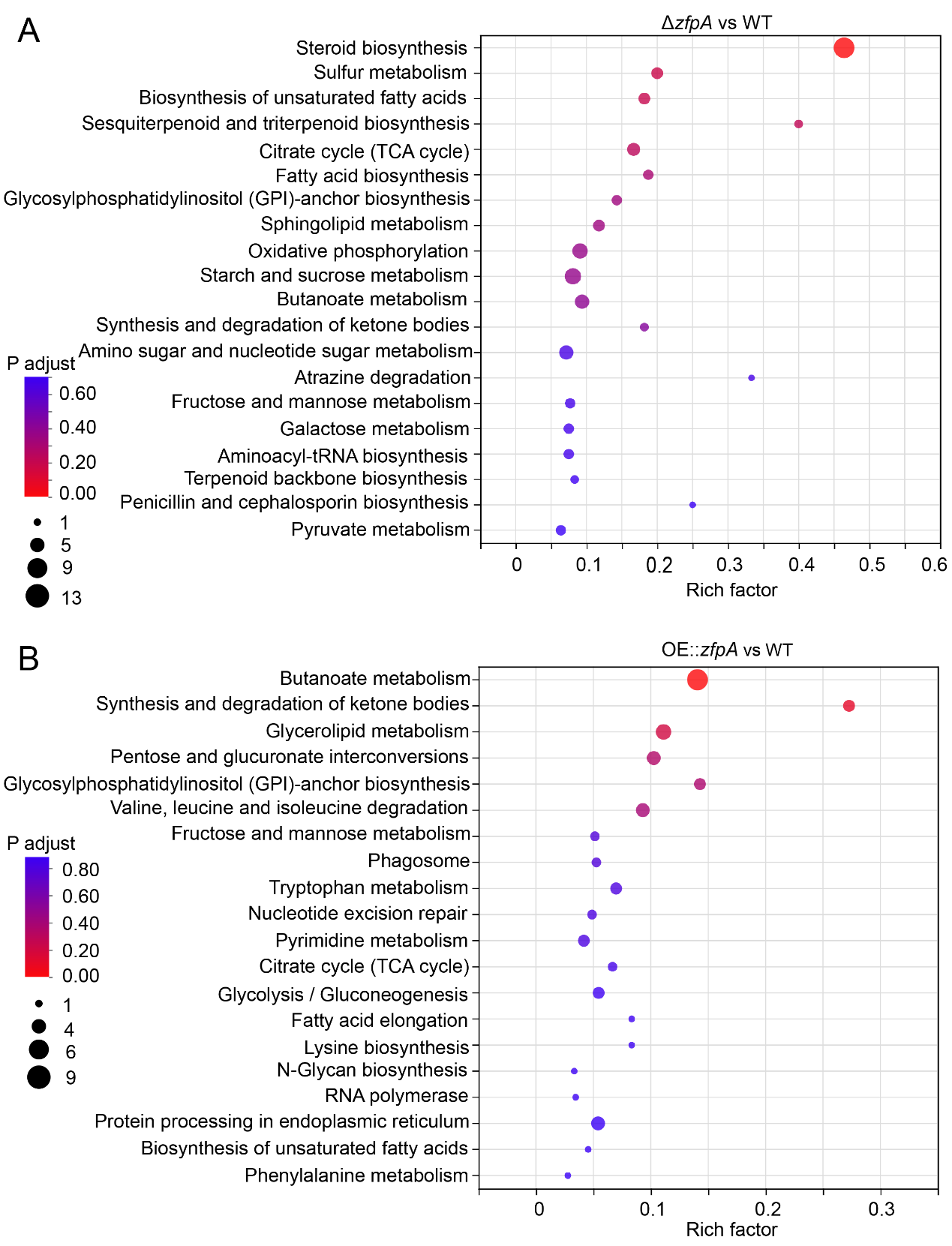


Fig. S8 Gene Ontology analysis down-regulated genes in the *zfpA* deletion (A) and *zfpA* overexpression strain (B).

Table S1. *A. fumigatus* strains used in this study

| Strains | Genotyppe |
| --- | --- |
| A1160 | *Δaku80; pyrG1* |
| *ΔzfpA* | *Δaku80; pyrG1; ΔzfpA::pyr4* |
| *zfpA^C^* | *Δaku80; pyrG1; ΔzfpA::pyr4; zfpA::hph* |
| *OEzfpA* | *Δaku80; pyrG1; gpdA(p)::zfpA:: pyr4* |
| WT*^zfpA-GFP^* | *Δaku80; pyrG1; zfpA::gfp::pyr4* |
| WT*^PatrF-lacZ^* | *Δaku80; pyrG1; AfatrF(p)::lacZ::hph* |
| *ΔzfpA^PatrF-lacZ^* | *Δaku80; pyrG1; ΔzfpA::pyr4; Afmdr1(p)::lacZ::hph* |
| *ΔatrF* | *Δaku80; pyrG1; ΔatrF::pyr4* |
| *ΔzfpAΔatrF* | *Δaku80; pyrG1; ΔzfpA::hph; ΔatrF:: pyr4* |
| *ΔzfpA ^Tet-crzA^* | *Δaku80; pyrG1; ΔatrF::pyr4; Tet-crzA::ptrA* |
| ZfpA^H272A^ | *Δaku80; pyrG1; ΔzfpA::pyr4; zfpA^H272A^; hph* |
| ZfpA^C284A^ | *Δaku80; pyrG1; ΔzfpA::pyr4; zfpA^C284A^; hph* |
| ZfpA^H305A^ | *Δaku80; pyrG1; ΔzfpA::pyr4; zfpA^H305A^; hph* |
| ZfpA^C316A^ | *Δaku80; pyrG1; ΔzfpA::pyr4; zfpA^C316A^; hph* |
| ZfpA^H339A^ | *Δaku80; pyrG1; ΔzfpA::pyr4; zfpA^H339A^; hph* |
| ZfpA^C377A^ | *Δaku80; pyrG1; ΔzfpA::pyr4; zfpA^C377A^; hph* |
| *ΔflbD* | *Δaku80; pyrG1; ΔflbD::pyr4* |
| *ΔAFUB_027970* | From Michael J. Bromley’s lab |
| *ΔAFUB_035140* | From Michael J. Bromley’s lab |
| *ΔAFUB_042120* | From Michael J. Bromley’s lab |
| *ΔAFUB_083250* | From Michael J. Bromley’s lab |

Table S2. Primers used in this study

| Name | Sequence (5 to 3) |
| --- | --- |
| zfpA p1 | CGGCGACCTAAAACACTGG |
| zfpA p2 | ATCTCCACTTCCCCTGTT |
| zfpA p3 | CGATTAAGTTGGGTAACGCCA GATGGCTTCTTTGTGCGC |
| zfpA p4 | ATAAGTAGCCAGTTCCCGAAAGC ATGCGGCCGCCTTCCCTCG |
| zfpA p5 | GTCTGTTTGACCCCGAGTC |
| zfpA p6 | GTCAATGAGAGGCTGCCAG |
| zfpA-not1 F | AAGGGCAATTCGCGGCCATCTCCACTTCCCCTGTTCCCCCTAG |
| zfpA-not1 R | CGAATTGAATTTAGCGGCC CCATTCATGATGCAGCATATGCA |
| zfpA-cla1 F | GAACCTTTAATCAAGCTT ATGTTGGCATTGGACTCGTCACG |
| zfpA-cla1 R | GAGGTCGACGGTATCGAT TCATGACATCAAGTCATCGATAT |
| zfpA-gfp p1 | AGGGAACCATGGTGTGTGG |
| zfpA-gfp p2 | GCTTCAACAGAGCGGATG |
| zfpA-gfp p3 | CCAGCGCCTGCACCAGCTCCTGACATCAAGTCATCGATATCA |
| zfpA-gfp p4 | CATCAGTGCCTCCTCTCAGACAGATGCGGCCGCCTTCCCTCG |
| zfpA-gfp p5 | CCAGAGTTGATTATGGAGAG |
| zfpA-gfp p6 | GTCTGTTTGACCCCGAGTC |
| zfpA-flag p3 | CATTCCCGGGGATCCCTCGAGTGACATCAAGTCATCGATATCA |
| zfpA-flag p4 | ATAAGTAGCCAGTTCCCGAAAGC ATGCGGCCGCCTTCCCTCG |
| EMSA-atrF L | AGCACGTGGTCGAAAGCCGTCCTAGTCAACCAGG |
| EMSA-atrF R | AGCACGTGGTCGAAAGCGCTGTTTGATGCGTATA |
| RT-atrB F | CTGGCCTCGACGGTCAATCC |
| RT-atrB R | TTGGCCAACAGCAACAGGGT |
| RT-atrF F | GCCGTCGTTCACCGTCATCT |
| RT-atrF R | AAGACCGGGTAGTCGCGGTA |
| RT-mdr4 F | ACGGCAACTGGTGAGAAGGC |
| RT-mdr4 R | CGAACTGCCCGCGTTGAATG |
